# Single-Phase Grounding Fault Types Identification Based on Multi-Feature Transformation and Fusion

**DOI:** 10.3390/s22093521

**Published:** 2022-05-05

**Authors:** Min Fan, Jialu Xia, Xinyu Meng, Ke Zhang

**Affiliations:** College of Automation, Chongqing University, Chongqing 400044, China; 202013131143@cqu.edu.cn (J.X.); 20164188@cqu.edu.cn (X.M.); smeta@163.com (K.Z.)

**Keywords:** single-phase grounding fault, multi-feature fusion, fault identification, feature transformation, deep learning

## Abstract

The frequent occurrence of single-phase grounding faults affects the reliable operation of power systems. When a single-phase grounding fault occurs, it is difficult to accurately identify the fault type because of the weak characterization and subtle distinction between different fault types. Therefore, this paper proposes a single-phase grounding fault type identification method based on the multi-feature transformation and fusion. Firstly, the Hilbert–Huang transform (HHT) was used to preprocess the fault recorded wave data to highlight the characteristics between different fault types. Secondly, the deep learning model ResNet18 and the long short-term memory (LSTM) are designed to extract the complex abstract features and time-series correlation features from the preprocessed data set separately. Finally, it designs a fusion model to combine the advantages of heterogeneous models to identify the type of single-phase grounding fault. Experiments validate that the method is good at fully mining the characteristics of the fault types contained in the fault recorded wave data, so it can identify multiple types of faults with strong robustness and provide a reliable basis for the subsequent formulation of targeted fault-handling measures.

## 1. Introduction

The basic requirements for power system operation are safety, reliability, high quality, and economy. Moreover, the safe and reliable operation of a power system is related to most aspects of national production and people’s lives.

The small current grounding system is widely used in 6–66 kV low and medium voltage distribution networks. This includes the neutral un-rounded power system, the neutral resonant grounded power system and the neutral resister grounded power system [1]. Single-phase grounding faults in the small current grounding system are the most common faults in the distribution system, accounting for approximately 80% of the total number of faults. 

When a single-phase ground fault occurs, a high-impedance loop is formed by the line-to-ground capacitance, and the short-circuit current is very small. Simultaneously, the line voltage remains symmetrical, which will not have a certain impact on the continuous power supply of the load in a short time, and it can run for 1–2 h with faults [1]. 

**Figure 1:**
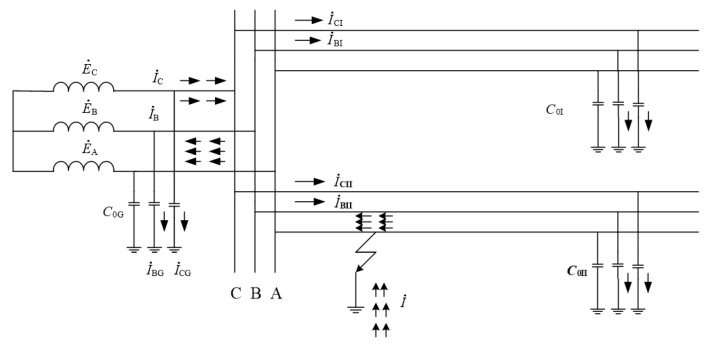
Capacitor current distribution diagram.

However, when a single-phase ground fault occurs, the phase-to-phase voltages of non-faulted phases rises to $\sqrt{3}$ times higher than the original. Figure 1 shows the capacitive current distribution diagram when a single-phase grounding fault occurs in an neutral un-rounded power system. It can be seen from the figure that a single-phase grounding fault occurs in phase A of line Ⅱ. The current direction of the faulty phase is from the line to the generatrix, and the current direction of normal phases is from the generatrix to the line.

**Figure 2:**
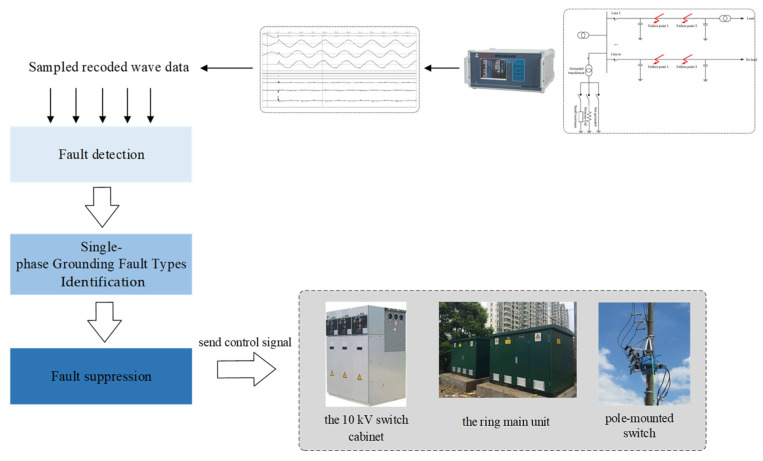
Diagnosis flow chart of single-phase grounding fault.

Therefore, if the power grid is still running for a long time with single-phase ground fault, it will create a threat to the weak point of the insulation in the power system, and even damage the equipment and disrupt the safe operation of the system. Furthermore, it may bring about huge economic losses and negative social impacts. Thus, it is necessary to accurately identify the fault type the first time, and provide a reliable basis for arranging targeted fault handling measures. The flow chart from the detection of single-phase grounding fault to fault handling is shown in Figure 2.

At present, there are many studies on the identification of single-phase ground fault types. According to the different methods of the application of characteristics, the existing identification methods can be divided into two categories: one is the characteristic analysis method [2,3,4,5,6,7,8,9,10,11,12,13,14,15] such as: Zhou et al. [5] proposes an online monitoring and identification method for high-resistance grounding faults in distribution networks, using high-order harmonics generated during high-resistance grounding faults as the judgement basis. Shen et al. [6] proposed a method for identifying single-phase arc grounding faults in distribution networks which proved that compared with metal grounding faults, arc grounding faults have a continuous transient process. The single-phase arc ground fault can be identified by extracting high-frequency signal slices. Chen et al. [8] proposed a high-resistance fault identification model based on the wavelet characteristic of the fault zero sequence variable. Chen et al. [11] analyzed the causes of high-resistance faults producing high-frequency components by using zero a sequence equivalent network, and proposed a single-phase high-resistance grounding fault identification method based on the energy ratio of the zero sequence voltage wavelet packet in the distribution network. Sheng et al. [14] proposed a high-resistance ground fault identification method based on the difference between the neutral point current and the line zero sequence current projection. 

Another category of identification method is to combine the time–frequency decomposition with machine learning [16,17,18,19,20,21,22,23,24,25], such as: Nakho et al. [16] proposed a model combined discrete wavelet transform and k-nearest neighbor machine learning algorithm to detect and classify the high-impedance single-phase grounding faults. Tong et al. [19] proposed a method for analyzing intermittent arc overvoltage in low-resistance grounded systems using the Hilbert–Huang transform to extract the intrinsic mode functions component of the zero sequence voltage, and then using SVM to identify the intermittent arc ground faults. Zhang et al. [20] used wavelet analysis to extract the features from the phase voltage and current, and built an XGBoost model to identify the three states of the neutral point ungrounded system, namely: no fault, ground fault and zero line fault. Liang [23] proposed an intelligent diagnosis method for a single-phase ground fault based on the data collected by the PMU and the deep learning theory. However, this method requires a large amount of data to achieve high accuracy. Srikanth et al. [24] proposed a novel three-dimensional deep learning algorithm for the classification of power system faults. The method can identify single-phase and two-phase ground faults, but cannot identify the subdivision types of single-phase ground faults.

The existing research results have achieved a certain effect in single-phase ground fault identification. However, most of them only select part of the characteristics of the distribution network, that is, the unique attributes of a certain type of fault for analysis. As a result, the description of the fault information is insufficient. It can only identify a few specific fault types. Most of these methods lack universality and the types of single-phase grounding faults have not been classified comprehensively, which is not conducive enough for the operation personnel to arrange targeted fault treatment measures.

This paper considers the comprehensive identification of seven types of single-phase ground faults, including high-resistance ground faults, intermittent arc ground faults, etc., for which more comprehensive fault characteristics need to be extracted. Due to the high dimensionality of the fault data collected on site, the number of sampling points is large and simultaneously a typical multivariate time series. It contains a wealth of complex nonlinear characteristics and timing correlation characteristics that are strongly related to the fault type. Deep learning is good at automatically learning complex and useful features from high-dimensional data sets. It compares with many excellent manual feature extractors that have appeared in the past, including scale invariant feature transform (SIFT) [26,27], Gabor filter [28], and oriented gradient histogram (HOG) [29,30]. Deep learning models can learn the features of different properties and different levels by building different structures and adjusting the number of hidden layers, and it can directly implement an end-to-end task or extract abstract features for the downstream task [31].

In summary, this paper proposes a single-phase grounding fault types identification method based on multi-feature transformation and fusion. The innovation of this method includes the following parts:

- A time–frequency analysis method, Hilbert–Huang transform with strong adaptive capability, was used in the data preprocessing part and was helpful for extracting the transient characteristics of faults and highlighting the characteristics of different fault types.
- The deep learning models ResNet18 and LSTM were designed to extract the complex abstract features related to the fault type from preprocessed data set, including complex nonlinear features and timing correlation features.
- A single-phase ground fault type identification model was constructed based on the idea of model fusion, which combines the advantages of heterogeneous models to enhance the overall identification accuracy and robustness of the model.

The rest of this paper is organized as follows. In Section 2, we reviewed the principles of the related knowledge, including the Hilbert–Huang transform, Resnet18 and long short-term memory (LSTM). The single-phase grounding fault type identification method based on multi-feature transformation and fusion is introduced in Section 3. Section 4 focuses on the design and analysis of experiments. Finally, Section 5 elaborates the conclusion of this paper and the prospect of future work.

## 2. Related Works

### 2.1. Comparison of Related Works

We compare the advantages and disadvantages of some related works with the method proposed in this paper, and summarize them in Table 1.

The table shows that the method proposed in this paper has made innovations in data preprocessing, feature extraction and modeling. The method can accurately identify a variety of subdivision types of single-phase grounding faults, and has made an obvious breakthrough in the identification scope. Refined and accurate identification results can provide strong support for subsequent fault operation and maintenance.

### 2.2. Principles of the Related Knowledge

In the following, we will briefly review the related knowledge about the Hilbert–Huang transform as well as that about the deep learning models Resnet18 and LSTM.

#### 2.2.1. Principle of Hilbert–Huang Transform

Hilbert–Huang transform is an adaptive time–frequency analysis method, and the result reflects the law of the frequency domain characteristics of the signal changing with time. As an important means of analysis in the time–frequency domain, Hilbert–Huang transform not only absorbs the multi-resolution advantages of the wavelet transform, but also overcomes the difficulty of selecting the wavelet basis function in wavelet transform. It can reflect local features, which is helpful for extracting important features in complex fault signals [32,33].

Hilbert–Huang transform includes two processes: empirical mode decomposition (EMD) and Hilbert transformation. Empirical mode decomposition adaptively decomposes a complex signal into a series of intrinsic mode functions (IMFs). The instantaneous frequency of intrinsic mode functions at any point is meaningful. After empirical mode decomposition, the Hilbert transform is performed on each intrinsic mode functions, and then the instantaneous frequency and instantaneous amplitude of each intrinsic mode function can be obtained.

**Figure 3:**
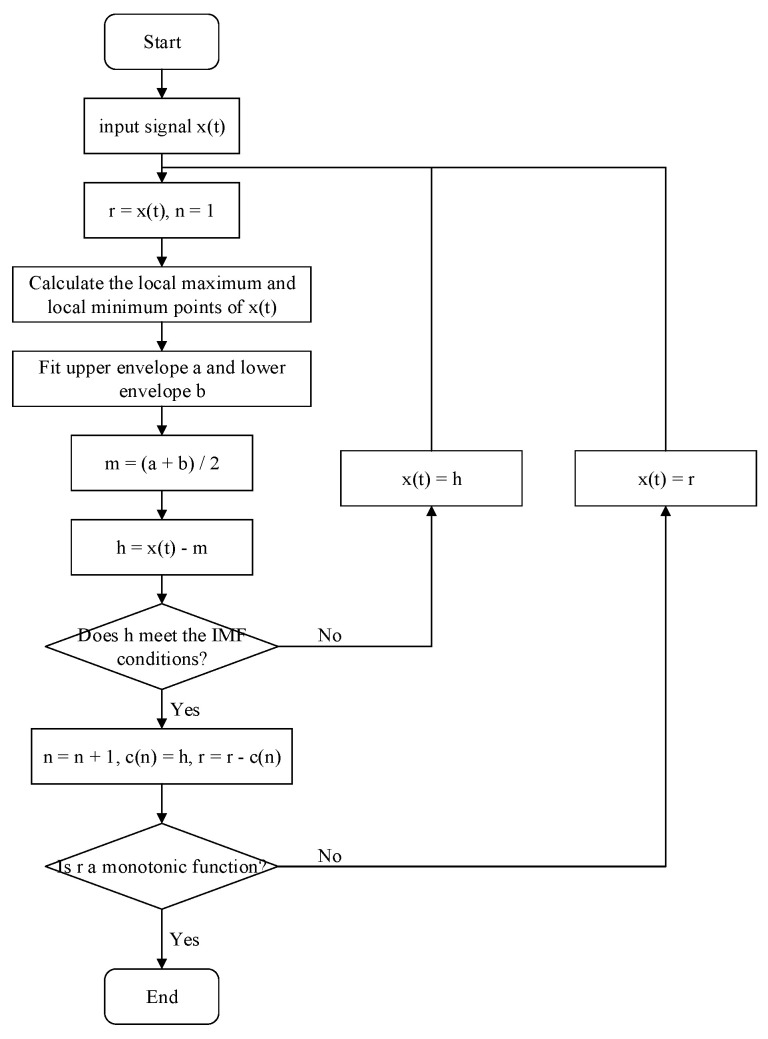
The process of empirical mode decomposition.

The intrinsic mode function components decomposed by empirical mode decomposition need to meet two conditions: one is that the number of extreme points and the number of zero-crossing points are the same or different by one in the entire signal length; and the other is that the upper and lower envelopes are symmetrical to the time axis at any time [34]. The process of empirical mode decomposition is shown in Figure 3.

The analytic signal $z_{i}\left(t\right)$ consists of the original intrinsic mode functions component$\;c_{i}\left(t\right)$ and its corresponding Hilbert transform result ${\hat{c}}_{i}\left(t\right)$.

(1) $$z_{i}\left(t\right)=c_{i}\left(t\right)+j{\hat{c}}_{i}\left(t\right)=A_{i}\left(t\right)e^{j\theta_{i}\left(t\right)}d\tau$$

The transformation process of ${\hat{c}}_{i}\left(t\right)\;$is shown in the following formula.

(2) $${\hat{c}}_{i}\left(t\right)=\frac{1}{\pi }\int_{-\infty }^{\infty }\frac{c_{i}\left(\tau \right)}{t-\tau }d\tau$$

The instantaneous amplitude $A_{i}\left(t\right)$ and instantaneous frequency $\omega_{i}\left(t\right)\;$from (1) are expressed as:

(3) $$A_{i}\left(t\right)=\sqrt{c_{i}^{2}\left(t\right)+{\hat{c}}_{i}^{2}\left(t\right)}$$

(4) $$tan\theta_{i}\left(t\right)=\frac{{\hat{c}}_{i}\left(t\right)}{c_{i}\left(t\right)}.\;$$

(5) $$\omega_{i}\left(t\right)=\frac{d\theta_{i}\left(t\right)}{dt}$$

The Hilbert spectrum of feature is expressed as:

(6) $$H\left(\omega ,t\right)=Re\left[\overset{q}{\underset{i=1}{\sum }}A_{i}\left(t\right)e^{j\int \omega_{i}\left(t\right)dt}\right]$$

where *q* represents the number of decomposed intrinsic mode function components. By performing a Hilbert–Huang transform on fault data, the hidden local transient features include instantaneous frequency, instantaneous amplitude and Hilbert spectrum are extracted.

#### 2.2.2. Principle of Resnet18 Network

The deep learning model learns more useful features by building the neural network model with many hidden layers, thereby ultimately improving the of classification or regression accuracy. The ability of deep learning models to extract features is mainly achieved through convolutional layers, activation operations, pooling layers, etc., of which the convolutional layer plays a major role. The convolution kernels in the convolution layer are equivalent to many different filters, and the feature extraction in the data set is obtained by using the convolution layer to “filter” the original features [35,36,37].

However, as the number of neural network layers increases, the problem of vanishing gradient restricts the performance of deep learning, and shallow networks hardly learn any knowledge. Additionally, because of the blindness of the convolution kernels parameters and the inhibitory effect of the activation function, the execution of each convolution operation and the corresponding activation operation will waste some information on the basis of the feature results extracted in the previous step.

**Figure 4:**
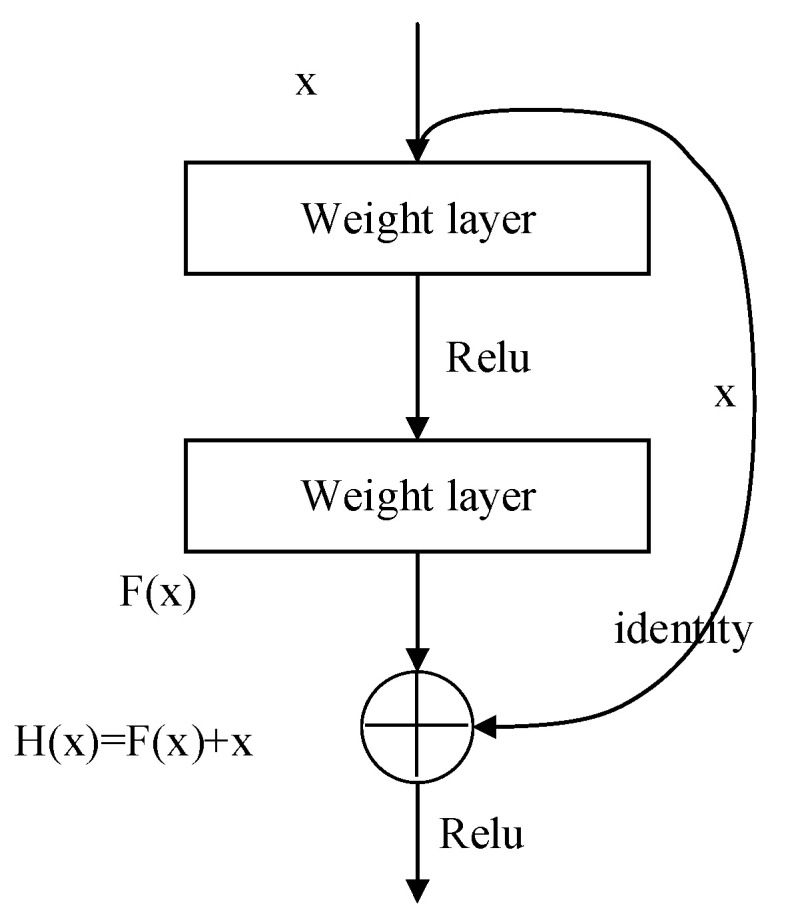
Residual network structure.

The Resnet network uses the residual connection method to solve the problem of the vanishing gradient in the deep network [38]. Its basic structure is shown in Figure 4.

By the way of the “shortcut connections”, the model directly passes the input *x* to the output, and the output F(x) after two convolutional layers is expressed as follows:

(7) $$F\left(x\right)=W_{2}\sigma \left(W_{1}x\right)$$

where σ represents the activation function (Relu). Additionally, the output result F(x) of the two convolutional layers is added to the identity mapping of the input *x* itself. The purpose of this operation is to ensure that the effect of the deep network is not weaker than that of the shallow network. Then, through the Relu function, the output y is obtained.

(8) $$y=F\left(x,\left\{W_{i}\right\}\right)+x$$

When the output of the residual block is required to be a specific dimension, a linear transformation $W_{s}$ can be performed on x in the shortcut connections, which is expressed as follows.

(9) $$y=F\left(x,\left\{W_{i}\right\}\right)+W_{s}x$$

Experiments have shown that the designed residual block usually needs to be at least two layers or more. If there are other convolutional layers or general layers between the two convolutional layers in the residual block, it can be expressed as:

(10) $$y=\overset{L-1}{\underset{i=1}{\sum }}F\left(x,\left\{W_{i}\right\}\right)+W_{s}x$$

In the process of backpropagating to solve the model parameters, the chain derivation process can be changed to the following form.

(11) $$\frac{dloss}{dx}=\frac{dloss}{dy}\times \frac{dy}{dx}=\frac{dloss}{dy}\left(1+\frac{d\sum_{i=1}^{L-1}F\left(x,\left\{W_{i}\right\}\right)}{dx}\right).\;$$

where 1 means that shortcut connections can inherit the gradient unconditionally. When the result of formula 12 is close to 0, the model can still maintain the gradient when the number of network layers is small. Therefore, the residual network can well solve the problem that the model is difficult to train due to the increase in the number of network layers [39].

(12) $$\mathrm{Gradient}=\frac{d\sum_{i=1}^{L-1}F\left(x,\left\{W_{i}\right\}\right)}{dx}$$

Therefore, this paper designs and uses the Resnet18 model to extract the complex abstract features of the data related to the single-phase ground fault type.

#### 2.2.3. Principle of Long Short-Term Memory (LSTM) Network

**Figure 5:**
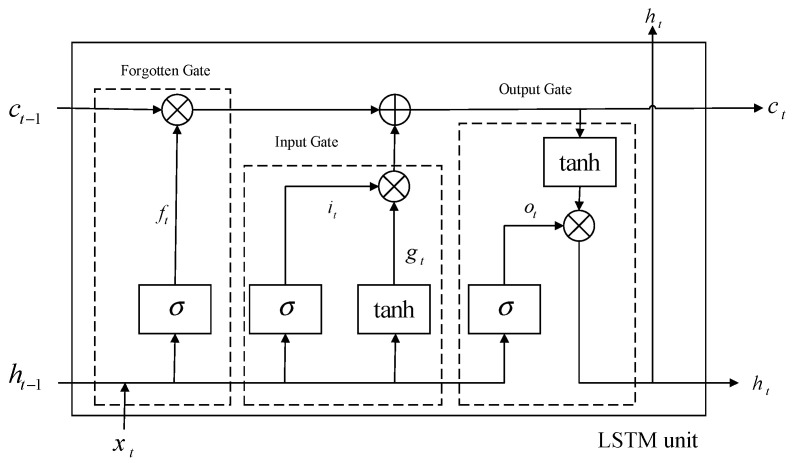
Basic structure of LSTM.

The LSTM network is a variant form of the recurrent neural network which can learn the long-term dependence information of time series. Its basic structure is shown in Figure 5.

**Figure 6:**
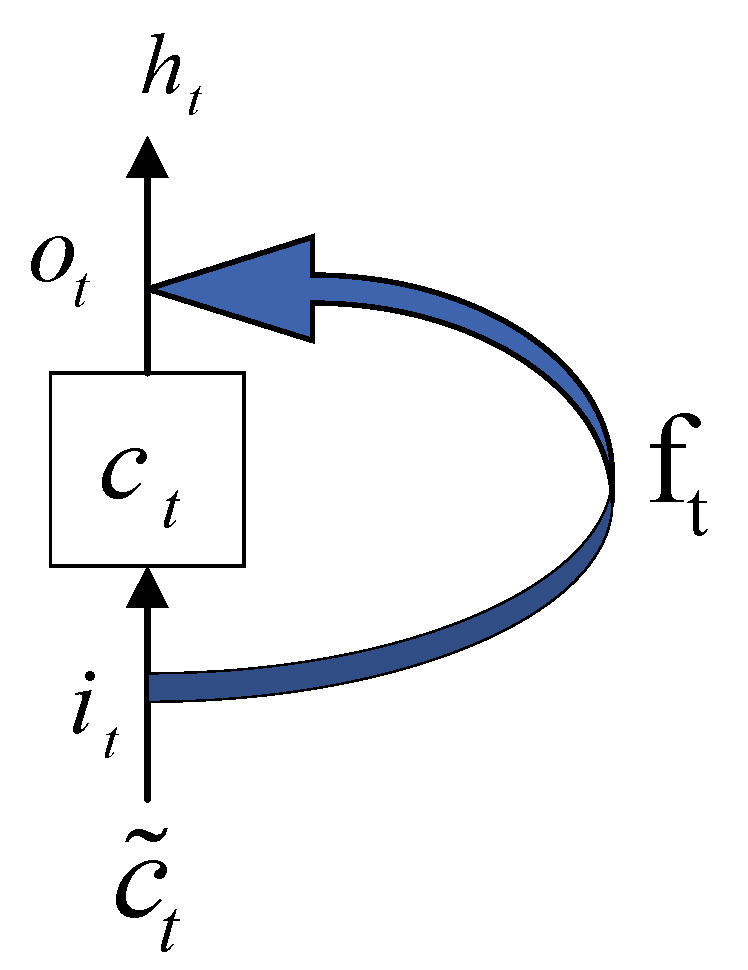
Calculation process of LSTM.

The LSTM model selectively memorizes the input information and retains the key information by controlling the input gate, the forget gate and the output gate [40]. The calculation process of LSTM is shown in Figure 6.

The calculation formulas of input gate$\;i_{t}$, forget gate$\;f_{t}$ and output gate$\;o_{t}$ are given below:

(13) $$i_{t}=\sigma \left(\omega_{i}\cdot \left[h_{t-1},x_{i}\right]+b_{i}\right)$$

(14) $$f_{t}=\sigma \left(\omega_{f}\cdot \left[h_{t-1},x_{i}\right]+b_{f}\right)$$

(15) $$o_{t}=\sigma \left(\omega_{o}\cdot \left[h_{t-1},x_{i}\right]+b_{o}\right)$$

Cell state (long-term memory) is expressed as:

(16) $$c_{t}=f_{t}\cdot c_{t-1}+i_{t}\cdot {\tilde{c}}_{t}$$

Memory (short-term memory) is expressed as:

(17) $$h_{t}=o_{t}\cdot \tanh\left(c_{t}\right)$$

The candidate state, that is, the new knowledge summed up, is expressed as:

(18) $${\tilde{c}}_{t}=\tanh\left(\omega_{c}\cdot \left[h_{t-1},x_{t}\right]+b_{c}\right).\;$$

where $i_{t},\;f_{t},\;o_{t}$ are the state results of the input gate, forget gate, and output gate, respectively.$\;\omega_{i},\;\omega_{f},\;\omega_{o}$ are the corresponding connection weights. $\;b_{i},\;b_{f},\;b_{o}$ are the corresponding bias items, and σ is the activation function.

Compared with other deep learning models, the loop structure network model can correlate the input of variables at different moments. It can extract more timing correlation characteristics than the general deep neural network model, and it is more suitable for the analysis of time series variables.

Since the recorded wave data of the distribution network is in the form of typical multivariable time series, it has a great correlation in time, so consider using the LSTM model to extract the timing correlation characteristics [41].

## 3. Problem Formulation and Methodology

**Figure 7:**
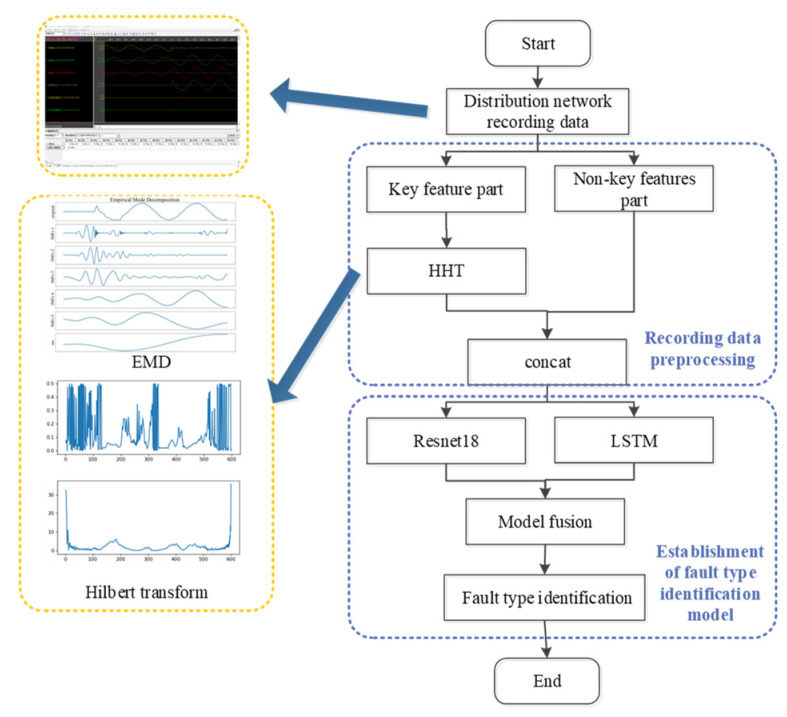
Overall process of the single-phase grounding fault type identification method.

The overall process of the single-phase grounding fault type identification method based on multi-feature transformation and fusion proposed in this paper is shown in Figure 7. The recorded wave data collected on the distribution network is an “electrocardiogram” of the real-time status and operation of the distribution network, which can provide the most direct and accurate evidence of the type identification of single-phase ground faults [42]. This paper therefore considers the fault recorded wave data as the original data set. Firstly, the fault recorded wave data are divided into the key feature part and the non-key feature part, and perform the Hilbert–Huang transform on the key feature part. As such, the important features related to the fault type are separated from the complex signal to generate new features. At the same time, in order to ensure the integrity of the fault information, the Hilbert–Huang transform result of the key feature part is spliced with the original non-key feature part; then, two deep learning models are designed and used: ResNet18 and LSTM models to learn the preprocessed data set, and based on the idea of model fusion, to fuse the learning results of the two deep learning models in order to identify the fault type.

### 3.1. Data Preprocessing

In order to clarify the observation object and reduce the amount of data analyzed, only the recorded wave data in fault state are intercepted for analysis.

For all the original features contained in the recorded wave data, since many of the characteristics remain constant throughout the entire period, or show periodic regular changes, it is of little significance for the identification of single-phase ground fault types. Based on the experience of the operation personnel, all electrical characteristics contained in the recorded wave data are divided into two parts: the key feature part and the non-key feature part. In order to further analyze the key features, we performed the Hilbert–Huang transform on them to obtain the corresponding time–frequency information. The data preprocessing part involved the sparse representation of compressed sensing technology. The original signal was transformed into a compressible time–frequency domain by Hilbert–Huang transform, and a sparse representation of the original signal was obtained. While reducing the feature dimension, its representation ability was improved.

The zero sequence current 3$I_{0}$ of the line bus is one of the key features. Perform empirical mode decomposition on 3$I_{0}$ of several lines with different fault types and compare the results.

**Figure 8:**
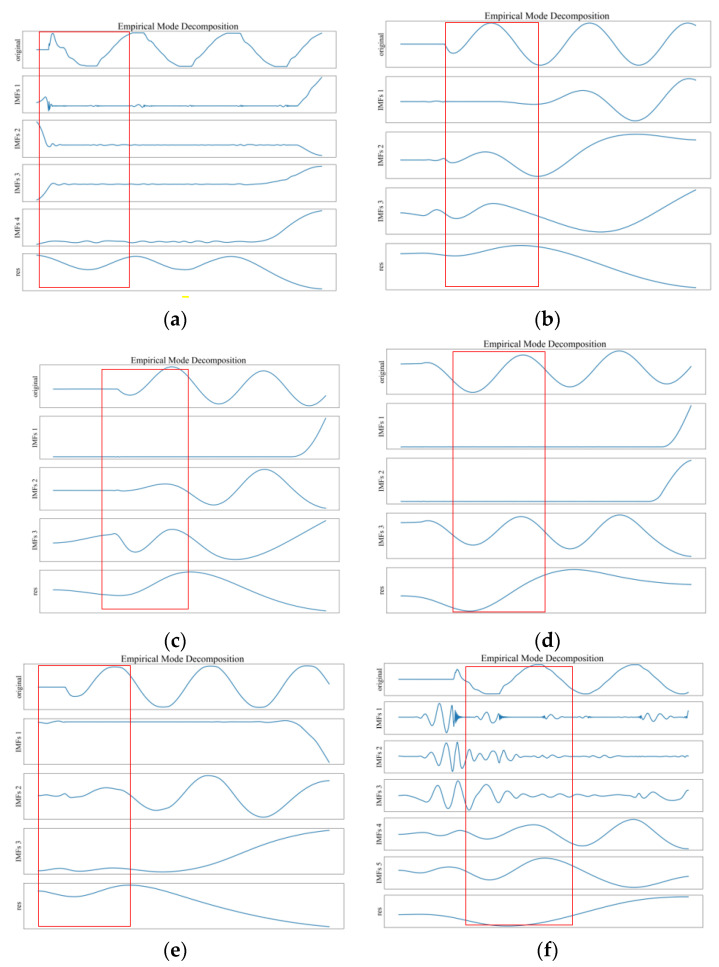
Comparison of empirical mode decomposition results.

In Figure 8, the first signal in each subgraph is the original signal, followed by imf1, imf2, …, imfn are the decomposed intrinsic mode function components, and the res represents the residual signal. The red rectangle emphasizes the transition stage from the normal state to the fault state. Since the empirical mode decomposition is adaptive, the number of decomposed intrinsic mode functions components is different. In Figure 8, (a) is an intermittent arc ground fault; (b) is a ground fault through a 250 Ω resistance; (c) is a ground fault through a 1000 Ω resistance; (d) is a ground fault through a 2000 Ω resistance; (e) is a soil ground fault; and (f) is a ground fault through arc resistance.

Figure 8 shows that there are obvious differences in the empirical mode decomposition results of the zero-sequence current 3$I_{0}.$ These zero-sequence current 3$I_{0}\;$come from the line bus with different fault types. The number of intrinsic mode function components, waveforms, and residual waveforms are not the same: these can be used as the basis for subsequent fault identification model research.

**Figure 9:**
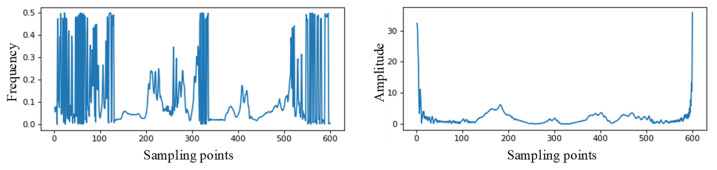
Instantaneous frequency and instantaneous amplitude of intrinsic mode function 1 (IMF1).

The Hilbert transform was performed on intrinsic mode function 1 of the line with intermittent arc ground fault, as shown by (a) in Figure 8. The instantaneous amplitude and instantaneous frequency curves with the sampling points were obtained, as shown in Figure 9.

The above results show that the use of the Hilbert–Huang transform is beneficial to extract the transient characteristics of the recorded wave data and enhances the feature difference between the different types of single-phase grounding faults.

Although the correlation between the non-key features part and the single-phase ground fault type is weak, it still contains some fault-related information. We spliced the result of the key feature part after the Hilbert–Huang transform with the original non-key feature part. While keeping the feature dimension within an acceptable range, it also guarantees the integrity of the fault information.

In order to make the preprocessed data set become an image sample set, which is convenient for subsequent use of convolution operations to extract features, it is also necessary to normalize the features and convert them into 2D grayscale images.

### 3.2. Single-Phase Ground Fault-Type Identification Model

#### 3.2.1. The Overall Structure of the Model

**Figure 10:**
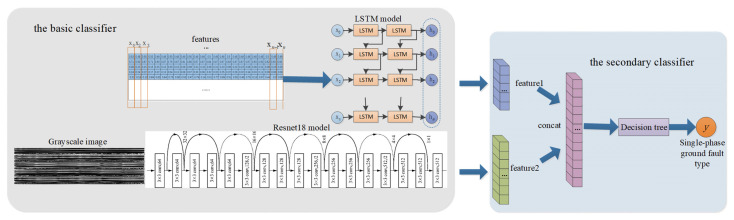
The Resnet18_LSTM_DT model framework.

The identification model proposed in this paper is Resnet18_LSTM_DT, and its framework is shown in Figure 10.

After the recorded wave data collected by the distribution network were preprocessed based on the operation described in Section 3.1, a hybrid model structure was then used to identify the type of single-phase grounding faults. The whole identification model structure consists of two parts: the basic classifier and the secondary classifier.

The preprocessed multi-dimensional time series data set was used to train the LSTM model, and the preprocessed data set was converted into 2D grayscale image, which was used to train the ResNet18 model. These two deep learning models were designed to extract the time-series-related features and complex nonlinear features of the data set, respectively. Then, the secondary classifier was used to fuse and learn the feature results extracted by the two basic learners, and finally identify the type of single-phase grounding fault.

#### 3.2.2. Extracting Complex Nonlinear Features Based on the Adjusted Resnet18 Model

**Figure 11:**
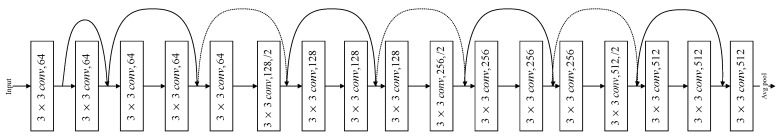
Basic structure of ResNet18.

According to the number of features and the hardware environment, choose the ResNet18 model to build one of the basic learners, and its structure is shown in Figure 11.

This paper only uses ResNet18 as the feature extractor. Combined with the characteristics of the fault recorded wave data, some changes were made to it on the basis of the original structure. In order to match the dimensionality of the fault recorded wave data, the convolution kernel of the first convolutional layer was modified to 3×3. Since the classification function of ResNet18 was needless at the end, the fully connected layer and the softmax layer were removed when the feature was to be extracted after this model was trained. The modified network parameters are shown in Table 2.

#### 3.2.3. Extracting Timing Correlation Features Based on the Adjusted LSTM Model

**Figure 12:**
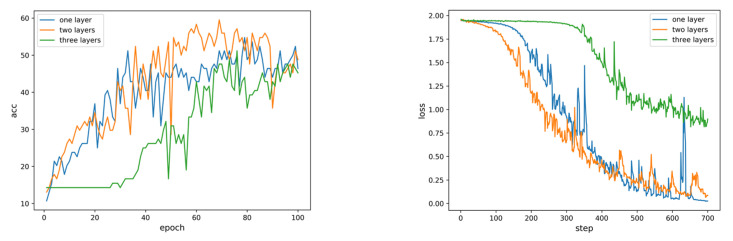
Comparison results of accuracy and loss function when LSTM sets different layers.

**Figure 13:**
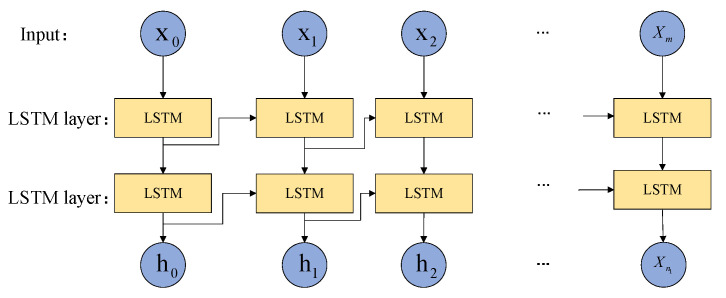
LSTM basic learner structure.

The model structure of LSTM was adjusted based on the fault recorded wave data and the experimental results of modifying the hyperparameters, as shown in Figure 12. When the number of LSTM layers was set to 2 and the number of nodes in the hidden layer was set to 100, the model had better accuracy and faster convergence speed. Therefore, the LSTM basic classifier built in this paper is shown in Figure 13, where $x_{i}$ is the multivariate feature vector at time i, m is the number of sampling time points, and $n_{1}\;$is the number of hidden nodes.

The two basic classifiers can be trained in parallel, and the loss functions are both cross-entropy loss functions.

#### 3.2.4. Construction of the Secondary Data Sets and Fusion Models

For the two basic learners built herein, the ResNet18 model can extract the complex nonlinear features of the data set, and the LSTM model can extract the time series-related features. The secondary learner fuses and learns the complementary feature results extracted by the two basic learners to generate the final classification result [43]. The secondary classifier selected in this paper is the decision tree model.

The decision tree algorithm can recursively select the optimal features of the sample, and segment the training data according to the feature, so that each subset of the data has a best classification result [44,45]. Moreover, its calculation is relatively simple, and no parameter assumptions are required. It is highly explanatory and easy to transform into classification rules as well as suitable for high-dimensional data [46].

The type of decision tree used in our methodology as the secondary classifier is the CART tree, which can handle continuous or discrete feature types. The criterion for selecting features is the Gini index. The calculation method of the Gini index is given below.

(19) $$G\left(p\right)=\overset{K}{\underset{k=1}{\sum }}p_{k}\left(1-p_{k}\right)=1-\overset{K}{\underset{k=1}{\sum }}p_{k}^{2}$$

The Gini index represents the probability of a randomly selected sample being incorrectly classified. The smaller the Gini index, the higher the purity of the separated subclass.

The output feature dimensions of LSTM and ResNet18 models are (1, $n_{1}$) and (1, $n_{2}$), respectively. The two sets of features are spliced and combined with the true label of the single-phase ground fault type (0–6, respectively, represent 7 different types of single-phase ground fault types) to form a secondary data set, as shown in Table 3.

The secondary classifier trained through the secondary data set. The grid search algorithm was used to find the best parameter settings of the CART model.

## 4. Analysis of Results

### 4.1. Establishment of Training Set and Testing Set

The recorded wave data used in this paper come from a real testing field in China. By changing the grounding mode of the neutral point, the type of grounding medium, the value of the grounding resistance, etc., different types of single-phase grounding fault recorded wave data can be obtained. Among them, the neutral point grounding method covers mainstream forms such as the neutral un-grounded, the neutral 32 resonant grounded, and the neutral resister grounded. The grounding medium includes common fault types such as intermittent arc grounding, stable arc grounding, earth grounding, and resistance grounding; typical values of grounding resistance are 250 Ω, 1000 Ω, 2000 Ω, and 5000 Ω. In the three grounding operation modes, the number of 420, 600, and 240 fault recorded wave data are generated, respectively. The sampling frequency of the recorded wave data device is 10 kHz, the sampling period includes 12,014 sampling points, and each recorded wave data sample contains 291 electrical characteristics. During the analysis process, the training set and the testing set were divided into a ratio of 8:2.

The experimental environment platform conditions used were those of the Windows10 X64 operating system, Inter i5-7200. The code was implemented using the Python programming language, ResNet18 and LSTM were implemented using the pytorch framework, and the classic machine learning algorithm used in the model fusion was implemented using the Keras framework.

### 4.2. Evaluation Index

In order to verify the identification performance of the model for single-phase grounding faults, the evaluation index used in this paper was the accuracy rate of the testing set, and the calculation formula is shown below:

(20) $$accuracy\left(y,\hat{y}\right)=\frac{1}{n_{samples}}\overset{n_{samples}-1}{\underset{i=0}{\sum }}1\left({\hat{y}}_{i}=y_{i}\right)$$

where $\hat{y}$ represents the identification category of the fault sample, y represents the true category of the fault sample, and $n_{samples}\;$is the number of testing samples.

### 4.3. Design and Analysis of Experiments

#### 4.3.1. Performance Comparison of Time–Frequency Analysis Methods

This experiment compares the identification performance of the model trained on data preprocessed by two time–frequency analysis methods, the Hilbert–Huang transform, and wavelet transform, respectively.

**Figure 14:**
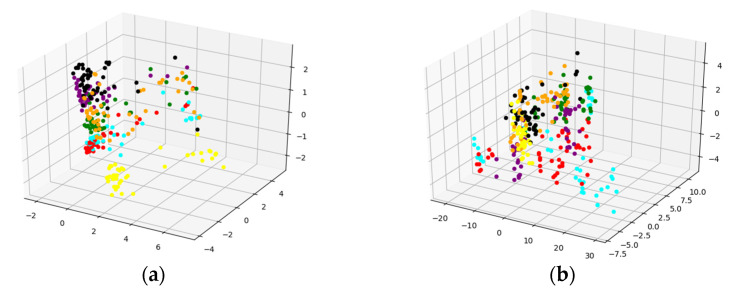
PCA visualization results: (**a**) Hilbert–Huang transform and (**b**) wavelet transform.

According to references [46,47,48], the wavelet basis function of the wavelet transform chose Daubechies (db5). The wavelet transform was performed on the key feature part and then spliced with the non-key feature part, and the single-phase ground fault type identification model was built in the same way. The data sets obtained by wavelet transform or Hilbert–Huang transform were used to train the identification model separately. The features extracted by the two basic learners were fused. Additionally, the dimensionality of the fusion result was reduced based on principal components analysis (PCA), and the visualization of the result are shown in Figure 14. It can be seen that compared to the sub-figure (b), the boundaries of each fault category in the sub-figure (a) are clearer, and the fused features can more accurately describe the fault type information. Although there are still some overlapping points between the different categories, the reason may be the inevitable loss of information caused by reducing the high-dimensional data to three-dimensional data.

**Figure 15:**
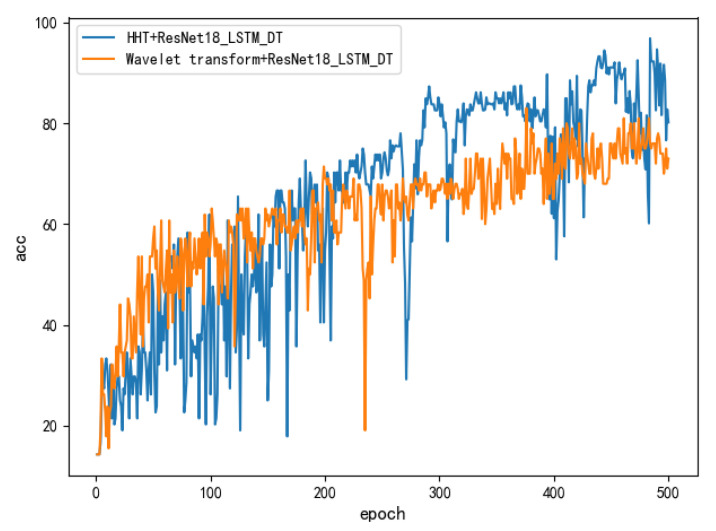
The hybrid model identification effect obtained by combining two time–frequency analysis methods, respectively.

The data sets preprocessed by these two time–frequency analysis methods were used to train the hybrid model ResNet18_LSTM_DT separately, and the final identification effect comparison is shown in Figure 15. This shows that the effect of the hybrid model obtained by combining with the Hilbert–Huang transform is significantly better than that of combining with the wavelet transform. After the model converges, their identification accuracy is 98.8% and 81.3%, respectively.

#### 4.3.2. Performance Analysis of the Preprocessing Methods

This experiment analyzed the effectiveness of Hilbert–Huang transform on the key features of the fault recorded wave data to improve the final classification accuracy of the model. The ResNet18-LSTM-DT hybrid model was trained by the data obtained by the three preprocessing methods, and then the recognition performance of the model was compared. The comparison results are shown in Table 4. The analysis shows that method b does not perform any feature preprocessing operations in advance, and that the classification accuracy is the lowest at 83.0%. In method a, the Hilbert–Huang transform is only performed on the key feature parts, and the remaining 286 original electrical quantities are directly discarded. It loses part of the fault-related information, but it decomposes and transforms the key features to extract new features that are more conducive to the identification of the fault type, so the classification accuracy is better than method b. The method c used in this paper combines the advantages of method a and method b. It extracts new features that are more conducive to fault type identification. Additionally, it also keeps the feature dimension within an acceptable range while ensuring the integrity of the fault information. The accuracy of fault type identification is also optimal.

#### 4.3.3. Performance Analysis of Fusion Process

**Figure 16:**
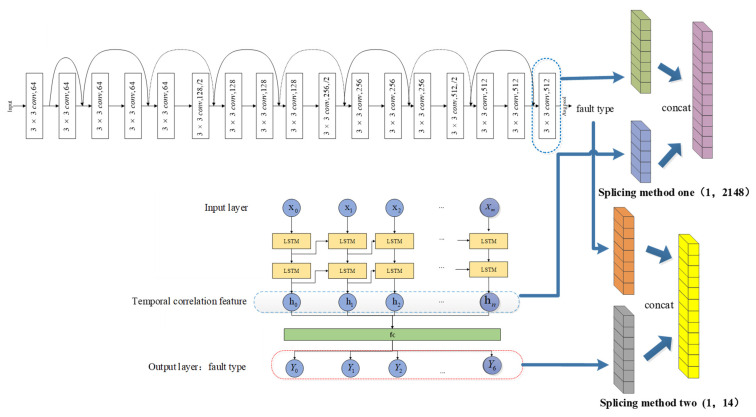
Feature splicing.

This experiment analyzed the relationship between the feature dimensions extracted by the basic learners and the final prediction classification accuracy. Additionally, it is verified that the decision tree algorithm used in the model fusion process has the highest classification accuracy. This experiment used two different feature splicing methods, as shown in Figure 16.

Splicing method 1: Two basic learners used the preprocessed data set for training, spliced the feature extraction results of the penultimate layer of the two models and the true type label of the single-phase ground fault to form the secondary data set. The feature dimension after splicing is (1, 2148).

Splicing method 2: Two basic learners used the preprocessed data set for training and performed single-phase grounding fault type identification. The two models outputted predicted classification results in the form of attribution probabilities of seven single-phase ground fault types. These two sets of classification probability and the true type labels of the single-phase grounding faults constituted the secondary data set. The feature dimension after splicing is (1, 14).

In this experiment, four machine learning algorithms were compared, namely: support vector machine, naive Bayes, logistic regression, and decision tree. They were separately used to train the secondary data set. Due to the small number of samples, in order to prevent the model from overfitting, the pruning operation was added when training the decision tree model, and the regularization term of the Logistic regression model was adjusted. The final classification accuracy results are shown in Table 5. The analysis shows that the prediction classification effect of the four machine learning models trained based on feature splicing method 1 are significantly better than that trained based on feature splicing method 2. Additionally, when using SVM and decision tree algorithms, there are few misclassifications. When using decision tree algorithms, the predictive classification effect is the best, with an accuracy of 98.8%. It is proved that the secondary data set obtained by the first feature splicing method can more fully express the information related to the single-phase ground fault type, and combining decision trees as secondary classifiers can achieve better predictive classification results.

#### 4.3.4. Performance Analysis of the Fusion Model and Single Model

This experiment compared and analyzed the prediction classification effect of the fusion model proposed in this paper and the effect of using the ResNet18 or LSTM model alone.

**Figure 17:**
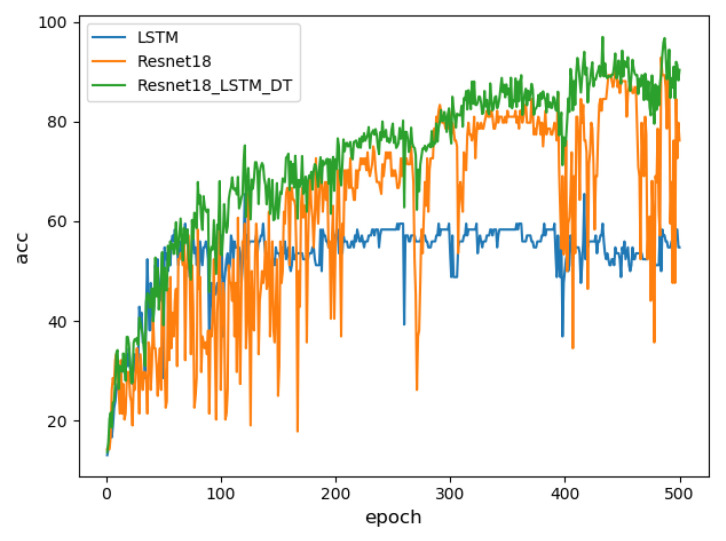
Comparison of the classification effect of using the hybrid model and using Resnet18 or LSTM alone.

The number of training epochs was set to 500, and the classification effect comparison of LSTM, ResNet18, and the hybrid model ResNet18_LSTM_DT is shown in Figure 17. It shows that the accuracy of using the LSTM model alone is the lowest. After 200 epochs, there is no significant increase, remaining at approximately 58%, but the fluctuations are relatively smooth after convergence. When using the ResNet18 model alone, because the model contains more parameters, the convergence speed of the model is slow. Moreover, the fluctuation of the model accuracy is relatively large, and the maximum change range of adjacent epochs is 46%. At the same time, due to the depth of the neural network and the huge amount of parameters, the ResNet18 model has a strong ability to fit the complex nonlinear relationship between the input and the output. After the model converges, the accuracy remains at approximately 90%. The hybrid model ResNet18_LSTM_DT performs the best in terms of model training speed, stability, and accuracy after model convergence. It combines the advantages of LSTM and ResNet18 to extract timing-related features and nonlinear features related to single-phase grounding fault type. After the model converges, the accuracy remains at approximately 98%, and at best can reach 98.8%. This experiment verifies the effectiveness of the hybrid model for the identification of the single-phase ground fault type.

#### 4.3.5. Comparison with AlexNet

As a classic CNN model, AlexNet is widely used in the field of image classification and recognition, and has achieved satisfactory results. Many tricks and methods in the AlexNet model have been used to this day, and it also provides ideas for the generation of subsequent classic neural networks.

Therefore, in order to verify the effectiveness of the Resnet18_LSTM_DT model in the identification of single-phase ground fault types, AlexNet was used for comparison. The same data set was fed to each network and the trained network was subsequently tested using the same testing set. Table 6 presents the testing results.

From the data in the above table, it is clear that the average accuracy of AlexNet for single-phase grounding fault type identification is 95.9%, while the average accuracy of the Resnet18_LSTM_DT model is 98.0%. Only in the 2000 Ω resistor ground fault did AlexNet work slightly better than the Resnet18_LSTM_DT model, and in other fault types, the identification accuracy of the Resnet18_LSTM_DT model is better than or equal to AlexNet.

#### 4.3.6. Robust Performance

The robustness of the model was verified by adding noise to the data set. This experiment directly added noise to the recorded wave data, and then used the methods mentioned in this paper to perform feature preprocessing and subsequent model training. The influence of noise on the single-phase ground fault identification method was analyzed by adding different types and different proportions of noise.

**Figure 18:**
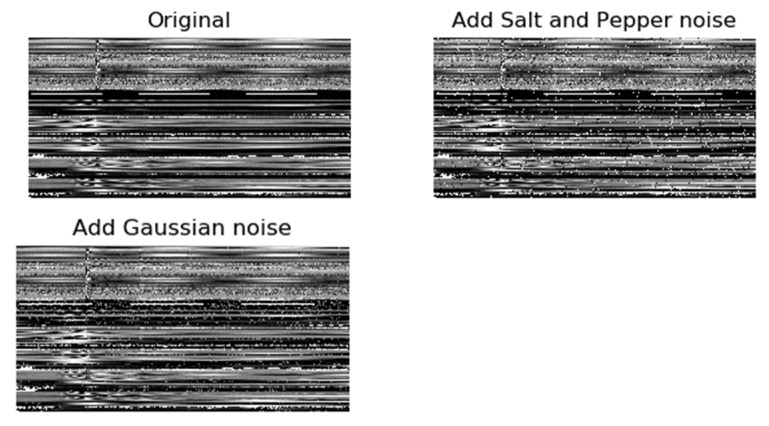
The data set is superimposed with salt and pepper noise or Gaussian noise.

The noise added in this experiment included two types: Gaussian noise and salt and pepper noise. Gaussian noise may originate from the noise and mutual influence of circuit components, while the salt and pepper noise usually is bright and dark noise generated by transmission channels and decoding processing. The average value of Gaussian noise generated in this experiment is 0. Different noise signals were generated by adjusting the noise ratio of salt and pepper noise and the standard deviation of Gaussian noise, which were superimposed with the recorded wave data collected by the distribution network. Among them, the results of adding salt and pepper noise with a noise ratio of 0.02 or Gaussian noise with standard deviation of 0.002 to the sample data are shown in Figure 18.

The effect of the identification model trained on the data set obtained by superimposing different noises is shown in Table 7. Although the classification effect of the model decreases with the increase in the noise ratio or standard deviation, the overall effect of the model is still relatively good, which proves that the model has a certain robust performance.

Through the analysis and demonstration of the above six experiments, it was proven that the single-phase grounding fault type identification method based on multi-feature transformation and fusion has advantages in feature preprocessing and model building. Combined with Hilbert–Huang transform, it alleviates the problems of weak fault characteristics and insignificant difference between the fault types when single-phase ground faults occur. The hybrid model ResNet18_LSTM_DT designed in this paper combines the advantages of each component model, can fully extract and utilize the information related to the fault type, and can accurately identify multiple types of single-phase grounding faults. Comparison experiments, ablation experiments, and noise experiments prove that this method has good accuracy, versatility, and robustness.

## 5. Conclusions and Future Work

This paper proposes a single-phase grounding fault type identification method based on multi-feature transformation and fusion. Firstly, this method used the Hilbert–Huang transform for the preprocessing of the recorded wave data of the distribution network to highlight the characteristics of different fault types; and then used LSTM and ResNet18 models to train the preprocessed data set; the decision tree model was used to fuse and learn the feature extraction results of the two basic classifiers to generate the final single-phase ground fault type identification result. Through the identification method proposed in this paper, the hidden features that reflect the difference of fault types are extracted and highlighted, and a variety of single-phase grounding fault types can be identified with high accuracy and robustness. In the application of the power system, the model trained well can be deployed in the 10 kV switch cabinet in the station, the ring main unit and sub-section post outside the station or pole-mounted switch. The control signal of the equipment was generated according to the model identification result and provided a reliable basis for the subsequent formulation of targeted fault handling measures.

In view of the single-phase grounding fault type identification method proposed in this paper, the following prospects are made:

- The method proposed in this paper is only based on the fault recorded wave data collected by the wave recorder for analysis. With the improvement of information collection devices and transmission systems in power systems, more characteristic data related to single-phase ground fault types can be obtained, such as: ledger data, attribute data, external data including environmental data and weather data, etc. It can also be included on the basis of fault type identification for analysis.
- The single-phase grounding fault has the problem of imbalance between the fault data and the normal data, and the imbalance between the various fault types, which will affect the classification accuracy of the identification model. Follow-up research may analyze and consider this.

## Figures and Tables

**Table 1:** Summary table of related works.

Author	Identification Scope	Advantages	Disadvantages	Ref.	Year of Publication
Jie Li et al.	High-impedance ground fault	Analyzing transient process when a high-impedance ground fault occurs.	1. Failure to identify and locate other types of single-phase ground faults. 2. The fault line-section location principle proposed in this paper does not consider the relationship between features and time series.	[3]	2020
Kangli Liu et al.	Fault feeder identification in flexible grounding system	The method combines wavelet packet transform and grey T-type correlation degree to achieve good recognition accuracy.	1. Wavelet transform needs to select a suitable mother wavelet and set a feasible number of decomposition layers, and the adaptive performance is insufficient. 2. The specific types of single-phase ground faults cannot be further divided.	[4]	2021
Yaru Sheng et al.	Fault with resonant grounded neutral	Solving the problem of difficulty in single-phase ground fault location under resonant grounding mode.	1. The robustness of the model is poor, for example, the parameter asymmetry will reduce the identification accuracy of the method. 2. The specific types of single-phase ground faults cannot be further divided.	[14]	2019
A. Nakho et al.	High-impedance ground fault	The method combines discrete wavelet transform with k-nearest neighbor machine learning algorithm to identification high-impedance ground fault.	1. The proposed approach puts forward high requirements for the advanced configuration and communication conditions of FIs. 2. Failure to identify and locate other types of single-phase ground faults.	[16]	2021
Pullabhatla Srikanth et al.	Two-phase and single-phase ground faults	The proposed network is novel and this method can identify the power system faults with high accuracy.	1. The memory cost of model increases compared to 2D CNN. 2. The specific types of single-phase ground faults cannot be further divided.	[25]	2021
Proposed method	Identification of seven types of single-phase ground faults, including high-resistance ground faults, intermittent arc ground faults, etc.	1. Hilbert–Huang transform is used to extract transient features of faults in data preprocessing, which is more adaptive than wavelet transform. 2. The deep learning models ResNet18 and LSTM are designed to extract complex nonlinear features and timing correlation features of preprocessed data, which increase the richness and completeness of fault information. 3. Based on the idea of model fusion, the method combines the advantages of heterogeneous models to enhance the overall identification accuracy and robustness.	1. The method is only based on the recorded wave data.	This work	

**Table 2:** Model parameters of ResNet18.

Name	Output Size	(Number of Channels, Core Size)
input	3 × 224 × 224	-
Conv1	64 × 112 × 112	64 × 3 × 3, step size = 2
the first piece: Conv2	256 × 56 × 56	$\left[\begin{array}{c} 64\;\times \;3\;\times \;3\\ 64\;\times \;3\;\times \;3 \end{array}\right]\times \;2$
the second piece: Conv3	512 × 28 × 28	$\left[\begin{array}{c} 128\;\times \;3\;\times \;3\\ 128\;\times \;3\;\times \;3 \end{array}\right]\times \;2$
the third piece: Conv4	1024 × 14 × 14	$\left[\begin{array}{c} 256\;\times \;3\;\times \;3\\ 256\;\times \;3\;\times \;3 \end{array}\right]\times \;2$
the fourth piece: Conv5	2048 × 7 × 7	$\left[\begin{array}{c} 512\;\times \;3\;\times \;3\\ 512\;\times \;3\;\times \;3 \end{array}\right]\times \;2$
ReLu	2048 × 7 × 7	-
average pool	2048 × 1	2048 × 7 × 7

**Table 3:** Format of the secondary data set.

	LSTM					Resnet18			label
Index	$h_{0}$	$h_{1}$	$h_{2}$	…	$h_{n_{1}}$	$h_{n_{1}+1}$	...	$h_{n_{1}+n_{2}}$	y
0	0.348	0.004	0.025	...	0.102	0.007	...	0.64	6

**Table 4:** Comparison of the identification effects of the hybrid model trained on the recorded wave data obtained by the three preprocessing methods.

	Recorded Wave Data Preprocessing Methods	Feature Dimension	Acc
Method a	Hilbert–Huang transform results of key features part	(185, 600)	0.879
Method b	No preprocessing	(291, 600)	0.830
Method c	Hilbert–Huang transform results of key features part + remaining original features	(471, 600)	**0.988**

**Table 5:** Comparison of the classification effects using different splicing methods and different secondary classifiers.

Classic Machine Learning Algorithms	SVM	Naive Bayes	Logistic Regression	Decision Tree
Splicing Method 1	0.979	0.935	0.863	**0.988**
Splicing Method 2	0.872	0.928	0.687	**0.908**

**Table 6:** Average correct identification rate of single-phase grounding fault types (%).

Fault Type	Resnet18_LSTM_DT	AlexNet
Intermittent arc grounding fault	**95.3**	92.2
Stable arc grounding fault	**100**	93.5
Earth grounding fault	**96.7**	94.0
Ground fault through 250 Ω resistor	**100**	**100**
Ground fault through 1000 Ω resistor	**100**	**100**
Ground fault through 2000 Ω resistor	97.8	**98.1**
Ground fault through 5000 Ω resistor	**96.2**	94.3
Average	**98.0**	95.9

**Table 7:** Comparison of the final classification effect of adding noise to the data set.

Noise Type	Noise Ratio/Standard Deviation	Acc
Salt and pepper noise	0.02	0.976
	0.05	0.969
	0.1	0.925
	0.2	0.871
	0.3	0.762
Gaussian noise	0.002	0.985
	0.005	0.967
	0.01	0.933
	0.02	0.857
	0.03	0.804

## Data Availability

The data presented in this study are available upon request from the corresponding author.

## References

[1] Zhang Z. (2014). Theoretical Research on Single-Phase Grounding Fault Line Selection in Small Current Grounding System.

[2] Elmitwally A., Ghanem A. (2021). Local current-based method for fault identification and location on series capacitor-compensated transmission line with different configurations. Int. J. Electr. Power Energy Syst..

[3] Li J., Wang G., Zeng D., Li H. (2020). High-impedance ground faulted line-section location method for a resonant grounding system based on the zero-sequence current’s declining periodic component. Int. J. Electr. Power Energy Syst..

[4] Liu K., Zhang S., Li B., Zhang C., Liu B., Jin H., Zhao J. (2021). Faulty Feeder Identification Based on Data Analysis and Similarity Comparison for Flexible Grounding System in Electric Distribution Networks. Sensors.

[5] Zhou F., Zhu R., Wang C., Wang B., Liu J. (2015). Online criterion and identification of single-phase ground fault with high resistence in distribution network. Chin. J. Sci. Instrum..

[6] Shen Y., Ruan L., Dai B., Liu N., Qiu L., He X. (2018). Identification of single-phase arc grounding fault in power distribution network. J. Wuhan Univ..

[7] Wang B., Geng J., Dong X. (2016). High-Impedance Fault Detection Based on Nonlinear Voltage-Current Characteristic Profile Identification. IEEE Trans. Smart Grid.

[8] Chen M., Hang Y., Zhai J. (2013). High impedance fault identification method of distribution network. J. Chongqing Univ..

[9] Ji P., Pei Y., Zhao S., Bai C., Wu B., Liang L., Pin D., Sun H., Zhi T. A Novel Location Method for Single-phase Grounding Fault for Distribution Network Based on Transient Technique. Proceedings of the 2018 Chinese Control And Decision Conference (CCDC).

[10] Zhou J., Ayhan B., Kwan C., Liang S., Lee W. (2012). High-Performance Arcing-Fault Location in Distribution Networks. IEEE Trans. Ind. Appl..

[11] Chen Z., Li Y., Peng H., Liu W. (2020). Identification of Single-phase High Resistance Earth Fault in Distribution Network Based on Wavelet Packet Energy Ratio of Zero Sequence Voltage. Sci. Technol. Eng..

[12] Shao W., Bai J., Cheng Y., Zhang Z., Li N. (2019). Research on a Faulty Line Selection Method Based on the Zero-Sequence Disturbance Power of Resonant Grounded Distribution Networks. Energies.

[13] Guo M., Liu S., Yang G. (2014). Grounding Faulty line detection based on transient waveform difference recognition for resonant earthed system. Electr. Power Autom. Equip..

[14] Sheng Y., Cong W., Bu X., Li X. (2019). Detection method of high impedance grounding fault based on differential current of zero-sequence current projection and neutral point current in low-resistance grounding system. Electr. Power Autom. Equip..

[15] Li Z., Ye Y., Ma X., Lin X., Ding C. (2020). Single-phase-to-ground fault section location in flexible resonant grounding distribution networks using soft open points. Int. J. Electr. Power Energy Syst..

[16] Nakho A., Hamam Y. Detection and Classification of Single Phase to Ground Faults Under High Resistance Ground Paths in Power Systems using Machine Learning. Proceedings of the 2021 Southern African Universities Power Engineering Conference/Robotics and Mechatronics/Pattern Recognition Association of South Africa (SAUPEC/RobMech/PRASA).

[17] Sun W., Paiva A., Xu P., Sundaram A., Braatz R.D. (2020). Fault detection and identification using Bayesian recurrent neural networks. Comput. Chem. Eng..

[18] Zhu J., Chen K., Peng Z., Zhang H., Wan X., Zhu D. (2020). Single-phase ground fault identification method for distribution network based on inception model and sample expansion. J. Phys. Conf. Ser..

[19] Tong Y., You Z., Shu L. (2012). Analysis of Intermittent Arc Overvoltages in Low Resistance Grounded Systems.

[20] Zhang J., Li Y., Zhang Z., Zeng Z., Cao Y. XGBoost Classifier for Fault Identification in Low Voltage Neutral Point Ungrounded System. Proceedings of the 2019 IEEE Sustainable Power and Energy Conference (iSPEC).

[21] Dong X., Shi S. (2011). Identifying Single-Phase-to-Ground Fault Feeder in Neutral Noneffectively Grounded Distribution System Using Wavelet Transform. Electr. Power Sci. Eng..

[22] Ni G., Bao H., Zhang L., Yang Y. (2010). Criterion Based on the Fault Component of Zero Sequence Current for Online Fault Location of Single-phase Fault in Distribution Network. Proc. CSEE.

[23] Liang J. Research on Rapid Diagnosis Method of Single-Phase Grounding Fault in Distribution Network Based on Deep Learning. Proceedings of the 2019 Chinese Automation Congress (CAC).

[24] Srikanth P., Koley C. (2021). A novel three-dimensional deep learning algorithm for classification of power system faults. Comput. Electr. Eng..

[25] Zhang L., Wang Y., Yan H., Shi F. Single-phase-to-ground Fault Diagnosis Based on Waveform Feature Extraction and Matrix Analysis. Proceedings of the 2019 9th International Conference on Power and Energy Systems (ICPES).

[26] Zhong B., Li Y. Image Feature Point Matching Based on Improved SIFT Algorithm. Proceedings of the 2019 IEEE 4th International Conference on Image, Vision and Computing (ICIVC).

[27] Zhang P., Shen L., Huang X., Xin Q. Application of an Improved SIFT algorithm in GPR images. Proceedings of the 2020 5th International Conference on Mechanical, Control and Computer Engineering (ICMCCE).

[28] Zhao M., Qiu W., Wen T., Liao T., Huang J. (2021). Feature extraction based on Gabor filter and Support Vector Machine classifier in defect analysis of Thermoelectric Cooler Component. Comput. Electr. Eng..

[29] Zhang T., Zhang X., Ke X., Liu C., Xu X., Zhan X., Wang C., Ahmad I. (2022). HOG-ShipCLSNet: A Novel Deep Learning Network With HOG Feature Fusion for SAR Ship Classification. IEEE Trans. Geosci. Remote Sens..

[30] Muhathir, Rizal R.A., Sihotang J.S., Gultom R. Comparison of SURF and HOG extraction in classifying the blood image of malaria parasites using SVM. Proceedings of the 2019 International Conference of Computer Science and Information Technology (ICoSNIKOM).

[31] Liu Z., Peng D., Zuo M.J., Xia J., Qin Y. (2021). Improved Hilbert–Huang transform with soft sifting stopping criterion and its application to fault diagnosis of wheelset bearings. ISA Trans..

[32] Shaik M., Shaik A.G., Yadav S.K. (2022). Hilbert-Huang transform and decision tree based islanding and fault recognition in renewable energy penetrated distribution system. Sustain. Energy Grids Netw..

[33] Mahata S., Shakya P., Babu N.R. (2021). A robust condition monitoring methodology for grinding wheel wear identification using Hilbert Huang transform. Precis. Eng..

[34] Wang Y., Xu H., Yi J., Liang S. (2020). Distribution Feeder Ground Fault Location Based on Hilbert-Huang Transform. J. Electr. Eng..

[35] Tan Y., Li Y., Liu H., Lu W., Xiao X. Performance Comparison of Data Classification based on Modern Convolutional Neural Network Architectures. Proceedings of the 2020 39th Chinese Control Conference (CCC).

[36] Zhang R., El-Gohary N. (2021). A deep neural network-based method for deep information extraction using transfer learning strategies to support automated compliance checking. Autom. Constr..

[37] Yu X., Wang S. (2019). Abnormality Diagnosis in Mammograms by Transfer Learning Based on ResNet18. Fundam. Inform..

[38] Zagoruyko S., Komodakis N. Wide Residual Networks. Proceedings of the 2016 British Machine Vision Conference.

[39] Lindemann B., Maschler B., Sahlab N., Weyrich M. (2021). A survey on anomaly detection for technical systems using LSTM networks. Comput. Ind..

[40] Shao Q., Guo L., Liu Y., Xie M., Dai C., Wang T., Zhang J., Cong Z. (2019). Identification Method for Single-phase Ground Fault of Distribution Network Based on LSTM Model. Guangdong Electr. Power.

[41] Yue Y., Li X., Zong Q. Development of automobile fault diagnosis expert system based on fault tree—Neural network ensamble. Proceedings of the 2011 International Conference on Electronics, Communications and Control (ICECC).

[42] Wu C., Wu K., Jiang M., Chen Q. (2007). Research on single-phase ground fault based on DSP and wavelet transform. Autom. Instrum..

[43] Sun B., Zhang H., Shi F. Machine Learning Based Fault Type Identification In the Active Distribution Network. Proceedings of the 2019 IEEE 3rd Information Technology, Networking, Electronic and Automation Control Conference (ITNEC).

[44] Saleh K., Ayad A. (2021). Fault zone identification and phase selection for microgrids using decision trees ensemble. Int. J. Electr. Power Energy Syst..

[45] Francis F.B., Ronald W., Peter N., Josephine N. A Modified Decision Tree and its Application to Assess Variable Importance. Proceedings of 2021 4th International Conference on Data Science and Information Technology (DSIT 2021).

[46] Zhang L., Liang Y., Sun Y., Xue Y., Li L., Jin X. Fault Type Recognition of Over-head Lines of Distribution Networks Based on Fault Indicator Waveform Data. Proceedings of the 2018 China International Conference on Electricity Distribution (CICED).

[47] Fan L., Yuan Z., Zhang K. (2011). Single-phase ground fault electrical model based on wavelet transform and its PSCAD/EMTDC simulation study. Power Syst. Prot. Control.

[48] Dong X., Li G., Jia Y., Xu K. (2021). Multiscale feature extraction from the perspective of graph for hob fault diagnosis using spectral graph wavelet transform combined with improved random forest. Measurement.

